# Downregulation of miR‑214-3p attenuates mesangial hypercellularity by targeting PTEN‑mediated JNK/c-Jun signaling in IgA nephropathy

**DOI:** 10.7150/ijbs.61274

**Published:** 2021-07-31

**Authors:** Yan Li, Ming Xia, Liang Peng, Haiyang Liu, Guochun Chen, Chang Wang, Du Yuan, Yu Liu, Hong Liu

**Affiliations:** Department of Nephrology, The Second Xiangya Hospital, Central South University, Hunan Key Laboratory of Kidney Disease and Blood Purification, Changsha, Hunan, China.

**Keywords:** IgA nephropathy, miR‑214-3p, mesangial cell proliferation, PTEN, JNK/c-Jun signaling

## Abstract

Mesangial cell (MC) proliferation and matrix expansion are basic pathological characteristics of IgA nephropathy (IgAN). However, the stepwise mechanism of MC proliferation and the exact set of related signaling molecules remain largely unclear. In this study, we found a significant upregulation of miR-214-3p in the renal cortex of IgAN mice by miRNA sequencing. *In situ* hybridization analysis showed that miR-214-3p expression was obviously elevated in MCs in the renal cortex in IgAN. Functionally, knockdown of miR-214-3p alleviated mesangial hypercellularity and renal lesions in IgAN mice. *In vitro*, the inhibition of miR-214-3p suppressed MC proliferation and arrested G1-S cell cycle pSrogression in IgAN. Mechanistically, a luciferase reporter assay verified PTEN as a direct target of miR-214-3p. Downregulation of miR-214-3p increased PTEN expression and reduced p-JNK and p-c-Jun levels, thereby inhibiting MC proliferation and ameliorating renal lesions in IgAN. Moreover, these changes could be attenuated by co-transfection with PTEN siRNA. Collectively, these results illustrated that miR-214-3p accelerated MC proliferation in IgAN by directly targeting PTEN to modulate JNK/c-Jun signaling. Therefore, miR-214-3p may represent a novel therapeutic target for IgAN.

## Introduction

Immunoglobulin A nephropathy (IgAN), first fully described by Berger and Hinglais in 1968 1, is the most common primary glomerulonephritis throughout the world. The incidence of IgAN and the risk of progression to end-stage renal disease (ESRD) in Asia are significantly higher than those in Europe and America 2. In China, IgAN accounts for 45.26% of primary glomerular diseases and is the most frequent cause (26.69%) of uremia 3. However, to date, the pathogenesis of IgAN has not been elucidated, nor has an effective treatment been established.

A 'multi-hit' hypothesis has been proposed to explain the development of IgAN. Specifically, galactose-deficient IgA1 (Gd-IgA1) is produced (Hit 1) and recognized by circulating antiglycan autoantibodies (Hit 2) to form immune complexes (Hit 3), which accumulate in the kidney and activate mesangial cells (MCs) (Hit 4) 4, 5. MCs are then activated to proliferate and secrete components of the extracellular matrix, cytokines and chemokines, resulting in podocyte and tubulointerstitial injury, glomerular sclerosis, and ultimately, progression to renal failure 5, 6. MC proliferation and increased synthesis of extracellular matrix are the basic pathological characteristics of IgAN. However, the stepwise mechanism of MC proliferation and the exact set of related signaling molecules remain to be fully clarified.

MicroRNAs (miRNAs) are a conserved class of small noncoding RNA molecules with a length of approximately 22 nucleotides that bind to the 3'-untranslated region (3'-UTR) of target mRNAs and repress gene expression at the posttranscriptional level 7. Accumulating evidence indicates that miRNAs are involved in kidney development, physiological function, and the pathogenesis of renal disease 8-11. As a result, therapeutic targeting of miRNA function through local or systemic delivery of miRNA mimics or inhibitors can be clinically translated for the treatment of various diseases 12. Although a series of studies have identified specific miRNAs that play crucial roles in IgAN, none have investigated the biological function of miRNAs in the proliferation of MCs in IgAN.

Phosphatase and tensin homolog (PTEN) is a tumor suppressor gene that participates in cellular proliferation, apoptosis, migration and invasion. Proximal tubule-specific deletion of PTEN in mice induced renal hypertrophy as a result of increased Akt signaling 13. c-Jun NH2-terminal kinase (JNK) is a member of the mitogen-activated protein kinase (MAPK) family that specifically catalyzes the phosphorylation of c-Jun to exert its biological activity. The JNK/c-Jun pathway has been identified as a functional target of the tumor suppressor PTEN 14, 15, and the activation of JNK/c-Jun promotes cell proliferation by accelerating G1-S cell cycle progression 16-18. Whether there is abnormal expression of PTEN in IgAN and whether MC proliferation is regulated by PTEN/JNK/c-Jun signaling remain to be further studied.

In this study, miRNA sequencing was performed to investigate the kidney expression of miRNAs in IgAN. We found that miR-214-3p was upregulated in MCs in IgAN. miR-214-3p functions by targeting PTEN to activate JNK/c-Jun signaling to promote MC proliferation. These results revealed novel mechanisms of MC proliferation and provided valuable targets and strategies for therapeutic intervention of IgAN.

## Materials and Methods

### Animals

Twenty-six six-week-old female BALB/c mice weighting 20 ± 2 g were obtained from Hunan SJA Laboratory Animal Co. Ltd. (Changsha, China) and were raised in a clean-grade room at ideal temperature and humidity. Mice were given free access to water and a standard laboratory diet. All animal experiments were conducted in accordance with guidelines approved by the Animal Care Ethics Committee of Xiangya Medical School, Central South University.

After one week of pre-feeding, mice were randomly divided into the following four groups: control group (control), IgAN group (IgAN), IgAN group treated with miR-214-3p antagomir (IgAN + miR-214-3p antagomir) and IgAN group infected with negative control (IgAN + antagomir NC). The IgAN mouse model was induced as previously described 19. BSA (Sigma) acidified water (800 mg/kg body weight) was administered by gavage every other day, combined with subcutaneous injection of CCl_4_ dissolved in castor oil (1:5; 0.1 ml) weekly and intraperitoneal injection (0.08 ml) biweekly. At weeks six and eight, LPS (Sigma) (50 μg) was injected through the tail vein. The IgAN mouse model was established at the end of the 11th week. During the process, one mouse in the IgAN model group and one in the control group were killed, and IgA deposition in the glomeruli was visualized by direct immunofluorescence to evaluate model establishment. For the IgAN + miR-214-3p antagomir and IgAN + antagomir NC groups, IgAN mice were subjected to tail intravenous injection of 20 mg/kg miR-214-3p antagomir or negative control every two weeks for 3 consecutive days from the sixth week. The other two groups received an equal amount of saline. Twenty-four-hour urine samples and renal tissues were collected carefully for subsequent experiments.

### Measurement of urinary albumin and creatinine levels

Urinary albumin and creatinine levels were measured using corresponding enzyme-linked immunosorbent assay kits (Exocell). All assays were performed according to the manufacturers' protocols. Proteinuria was reported as the ratio of urinary albumin to urinary creatinine (ACR).

### miRNA sequencing

The miRNA expression profiles of the renal cortex from control and IgAN mice (n =3) were obtained with the help of LC-BIO (Hangzhou, China). Total RNA was extracted with the TRIzol reagent (Invitrogen), and its quantity and purity were determined using the Agilent 2100 Bioanalyzer. Approximately 1 μg RNA and TruSeq Small RNA Sample Prep kits (Illumina) were used to prepare a small RNA library, following the manufacturer's instructions. We then completed sequencing with an Illumina HiSeq 2500 system. After various quality control processes, the unique sequences were retained and mapped to mouse precursors using a BLAST (Basic Local Alignment Search Tool) search in miRBase 21.0. The differentially expressed miRNAs were selected when the significance threshold between the two groups was <0.05.

### Cell culture and transfection

A mouse glomerular mesangial cell line (SV40 MES 13, Shanghai, China) was cultured in a 3:1 mixture of DMEM and Ham's F-12 medium, supplemented with 10% fetal bovine serum (FBS) and 14 mM HEPES (all from Gibco) at 37 °C in an atmosphere of 5% CO_2_. HEK-293T cells were obtained from the American Type Culture Collection (USA) and cultured in DMEM containing 10% FBS at 37 °C in 5% CO_2_. Monomeric human IgA1 (Abcam) was heated and aggregated at 65 °C for 150 minutes to obtain polymeric IgA1 (p-IgA1), as previously described 20. The miR-214-3p inhibitor, negative control (inhibitor NC), miR-214-3p mimic, negative control (mimic NC), PTEN siRNA and control siRNA were purchased from RiboBio (Guangzhou, China). All vectors were transfected with Lipofectamine 2000 (Invitrogen) according to the recommended protocol. After transfection, the cells were starved in serum-free medium for 12 hours, and then incubated with p-IgA1 (25 µg/ml) for 24 hours to construct the IgAN MC model 21.

### Cell proliferation assay

We used a Cell Counting Kit-8 (CCK-8) assay (Dojindo, Japan) to access cell proliferation. Cells were seeded in 96-well plates at a density of 5000 cells per well. After treatment, 10 μl CCK-8 solution was added to each well and incubated for 1.5 hours at 37 °C. Viable cell numbers were estimated by measuring the optical density (OD) at 450 nm.

### Cell cycle analysis

The treated cells were fixed with 70% ice‑cold ethanol overnight at 4 °C, and then stained with a mixture of propidium iodide and RNase A (Cell Cycle and Apoptosis Analysis Kit, Beyotime) for 30 minutes at 37 °C. All samples were analyzed using flow cytometry (BD Biosciences), and the distribution of cell cycle phases was measured using the Modfit Software. The proliferation index (PI) was calculated as the sum of the percentage of cells in S phase and G2/M phase.

### Luciferase reporter assay

The 3'-UTR of mouse PTEN mRNA containing wild-type or mutant miR-214-3p binding sites was cloned into the psiCHECK^TM^-2 vector (Promega). HEK-293T cells were co-transfected with luciferase reporter plasmid and the miR-214-3p mimic or negative control (mimic NC) using Lipofectamine 2000 (Invitrogen). After forty-eight hours, luciferase activity was determined using a Dual-Glo Luciferase Reporter Assay System (Promega) according to the manufacturer's instructions. The firefly luciferase activity of each sample was normalized to Renilla luciferase activity.

### Real-time PCR

Total RNA from MCs and renal cortex tissues was extracted with the TRIzol reagent (Invitrogen). mRNA reverse transcription was completed using the PrimeScript RT Reagent Kit with gDNA Eraser (TaKaRa). Real-time PCR was performed using SYBR Green Master mix (TaKaRa) on a LightCycler 96 System (Roche). For the quantification of miR-214-3p, stem-loop real-time PCR was adopted. The relative expressions levels of miR-214-3p and PTEN were normalized to those of the internal controls U6 (miR-214-3p) and GAPDH (PTEN). Each sample was shown as 2^-∆∆Ct^ values. All primers were purchased from Sangon (Shanghai, China).

### Immunoblot analysis

Renal cortex tissues or treated cells were harvested and lysed on ice using RIPA lysis buffer (Beyotime), which included protease and phosphatase inhibitors (Roche). The protein levels were measured using a BCA protein assay kit (Thermo Fisher Scientific). Equivalent amounts of protein were electrophoresed by SDS-PAGE and transferred to polyvinylidene difluoride membranes. After blocking in 5% bovine serum albumin for 1 hour at room temperature, membranes were incubated overnight at 4 °C with the following primary antibodies: PTEN (1:10000, Abcam), JNK (1:1000, Abcam), p-JNK (1:1000, Cell Signaling Technology), c-Jun (1:1000, Cell Signaling Technology), p-c-Jun (1:1000, Cell Signaling Technology), PCNA (1:2000, Proteintech), cyclinD1 (1:10000, Abcam), GAPDH (1:5000, Proteintech), and β-actin (1:2000, Servicebio). After incubation with corresponding horseradish peroxidase (HRP)-conjugated secondary antibodies at room temperature for 1 hour, antigens on the blots were visualized with an enhanced chemiluminescence kit (Millipore).

### Histology and immunohistochemical staining

Renal tissues were fixed in 4% paraformaldehyde, embedded in paraffin, and sliced into 4 μm sections. Hematoxylin-eosin (H&E) and periodic acid-Schiff (PAS) staining were conducted to access MC proliferation and matrix expansion. Immunohistochemical staining was performed according to standard protocols. Briefly, deparaffinized tissue sections were sequentially subjected to rehydration and antigen retrieval using heated citrate. After subsequent incubation in 3% H_2_O_2_ and blocking solution, the slides were exposed to anti-PTEN antibody (1:200, Proteintech) at 4 °C overnight and stained with HRP-linked secondary antibodies. The color was developed with a DAB kit (Vector Laboratories).

### Immunofluorescence

Immunofluorescence analysis of IgA deposition was conducted as previously described 22. Fluorescein isothiocyanate (FITC)-labeled goat anti-mouse IgA (1:50, Abcam) was used to detect IgA in renal tissues. Immunofluorescence staining in cultured cells was performed as follows. Cells were washed with phosphate-buffered saline (PBS), fixed with 4% paraformaldehyde and permeabilized with 0.1% Triton X-100. After being blocked with 5% bovine serum albumin, the cells were sequentially stained with anti-PTEN antibody (1:100, Proteintech) overnight at 4 °C, goat anti-mouse IgG conjugated with Alexa Fluor 488 (1:1000, Abcam) for 1 hour at room temperature, and DAPI (Beyotime). Samples were imaged using laser scanning confocal microscopy.

### *In situ* hybridization

*In situ* hybridization was performed as described previously 23. In brief, after deparaffinization and hydration, paraffin-embedded kidney tissue sections were incubated with 20 μg/ml proteinase K for permeabilization followed by a prehybridization solution at 37 °C for 1 hour. The specimens were then treated with digoxigenin-labeled mmu-miR-214-3p LNA probe (Servicebio) overnight at 37 °C. Following incubation with 5% BSA to remove nonspecific staining, the samples were probed with anti-digoxigenin-HRP for 1 hour at 37 °C. The signal was displayed by adding DAB solution and recorded using microscopy.

### Statistical analysis

Data are expressed as the mean ± SD of at least three independent experiments. Statistical differences between two groups were determined by the two-tailed Student's t-test, and differences in multiple groups were analyzed by one-way analysis of variance. *P* < 0.05 was considered statistically significant. All statistical analyses were performed using GraphPad Prism 7.0 and SPSS 24.0 statistical software.

## Results

### The expression of miR-214-3p was upregulated in IgAN MCs

**Figure 1:**
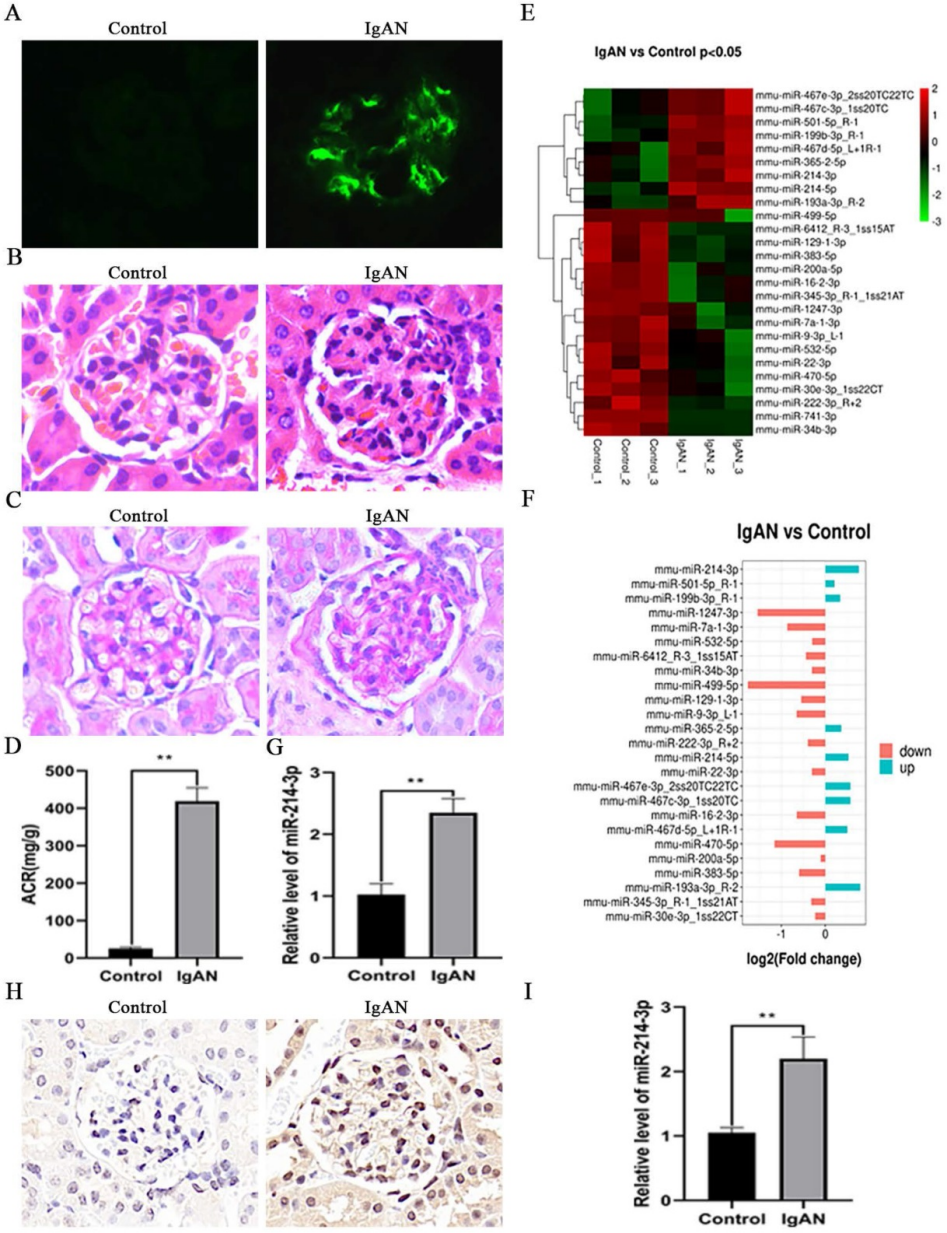
**miR-214-3p expression was upregulated in IgAN mesangial cells (MCs).** (A) Immunofluorescence staining of IgA in the glomeruli. Original magnification, 400x. (B-C) Representative images of hematoxylin and eosin (HE) staining and periodic acid-Schiff (PAS) staining in control and IgAN mice. Original magnification, 400x. (D) Proteinuria was reported as the ratio of urinary albumin to urinary creatinine (ACR). (E-F) Clustering map of miRNA expression in the renal cortex from control and IgAN mice (*P* < 0.05). (G) The differential expression of miR-214-3p in the renal cortex of control and IgAN mice was validated by real-time PCR. The relative level of miR-214-3p was normalized to the level of U6, and the ratio of control mice was arbitrarily set as 1. (H) *In situ* hybridization analysis showed that miR-214-3p was significantly elevated in MCs from the mouse renal cortex following IgAN. Original magnification, 400x. (I) The differential expression of miR-214-3p in MCs from the control and IgAN groups was validated by real-time PCR. The relative level of miR-214-3p was normalized to the expression of U6, and the ratio of control mice was arbitrarily set as 1. All data are expressed as the mean ± SD. *P < 0.05, **P < 0.01.

To investigate the potential role of miRNAs in the pathogenesis of IgAN, we initially established a mouse model of IgAN. Immunofluorescence staining indicated that the model groups had obvious IgA deposits in the glomeruli, while there was no IgA deposition in the control groups (Figure 1A). H&E and PAS staining showed that IgAN mice had pronounced mesangial hypercellularity compared with the control mice (Figure 1B-C). Proteinuria was also significantly elevated in IgAN mice (Figure 1D). All these results showed that the IgAN mouse model was successfully established. Then, we screened the differential miRNA expression profiles in the renal cortex of control and IgAN mice by miRNA sequencing. As illustrated in Figure 1E, 17 miRNAs were markedly upregulated, whereas 8 miRNAs were downregulated in IgAN mice compared with the control group (*P* < 0.05). Among these miRNAs, miR-214-3p was obviously elevated in IgAN mice (Figure 1F). We further performed real-time PCR on the isolated renal cortex to validate this sequencing result (Figure 1G). *In situ* hybridization analysis revealed that miR-214-3p expression was significantly increased in MCs from the renal cortex following IgAN (Figure 1H). Real-time PCR also confirmed an increase in miR-214-3p in MCs from IgAN mice (Figure 1I).

### Knockdown of miR-214-3p alleviated renal lesions in IgAN mice

**Figure 2:**
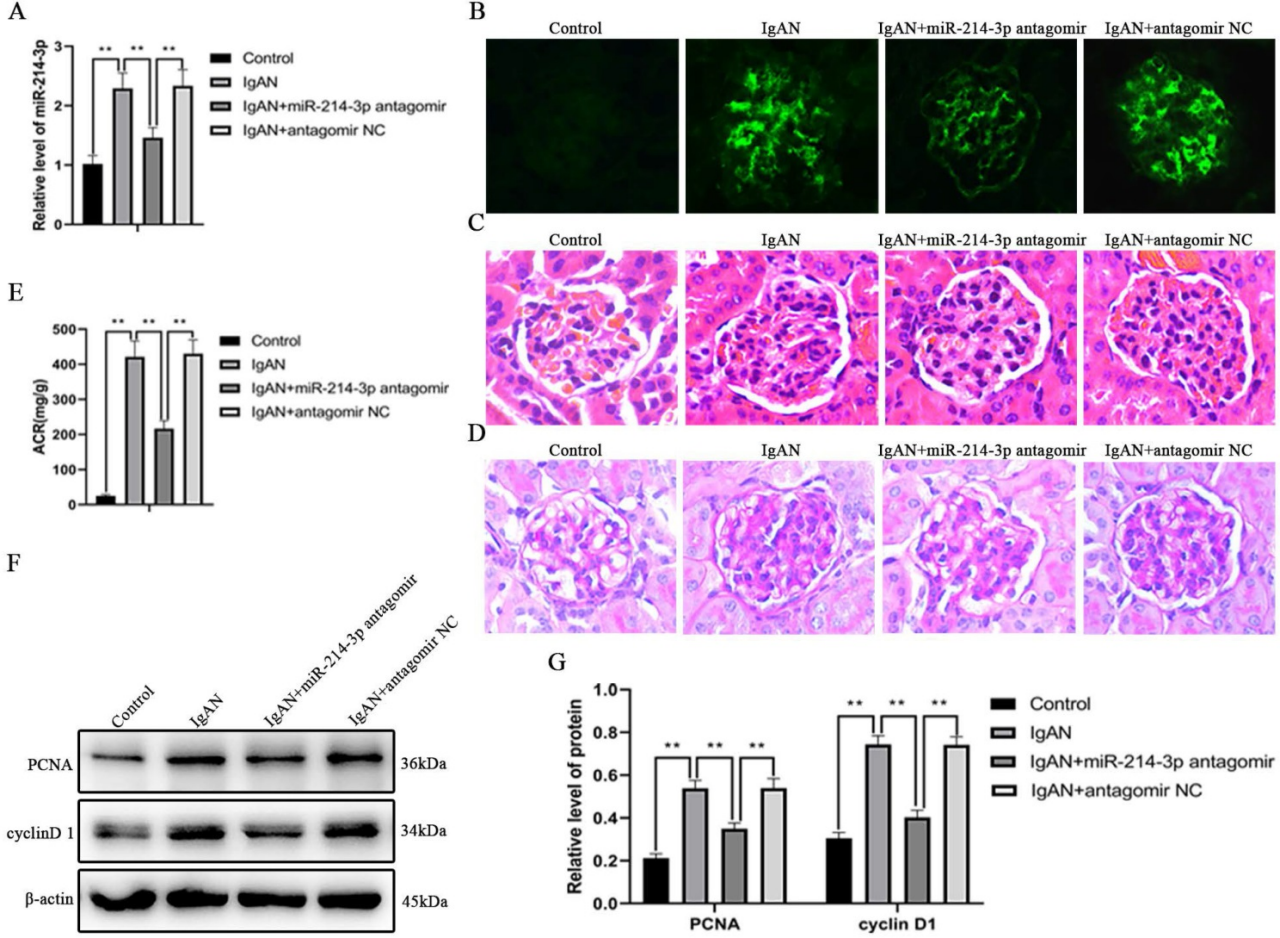
** Knockdown of miR-214-3p alleviated renal lesions in IgAN mice.** IgAN mice were subjected to tail intravenous injection of 20 mg/kg miR-214-3p antagomir or negative control (antagomir NC) every two weeks for 3 consecutive days from the sixth week to the eleventh week. (A) The expression of miR-214-3p in the renal cortex. The relative level of miR-214-3p was normalized to the expression of U6, and the ratio of control mice was arbitrarily set as 1. (B) Immunofluorescence staining of IgA in the glomeruli. Original magnification, 400x. (C-D) Representative images of hematoxylin and eosin (HE) staining and periodic acid-Schiff (PAS) staining. Original magnification, 400x. (E) Proteinuria was reported as the ratio of urinary albumin to urinary creatinine (ACR). (F) Immunoblot analyses and (G) quantitative determination of PCNA and cyclinD1 protein levels in the renal cortex. For densitometry, the signals of the target proteins were normalized to the β-actin signal of the same samples to determine the ratios. All data are expressed as the mean ± SD. *P < 0.05, **P < 0.01.

To examine the effect of miR-214-3p on IgAN *in vivo*, miR-214-3p antagomir or negative control were administered to IgAN mice. We confirmed that tail vein injection of miR-214-3p antagomir markedly reduced the level of miR-214-3p in kidney tissues (Figure 2A). Treatment with the miR-214-3p antagomir decreased IgA deposition in the glomeruli, ameliorated mesangial hypercellularity and improved proteinuria in IgAN mice (Figure 2B-E). Consistently, immunoblot analysis detected that the miR-214-3p antagomir inhibited the protein expression of PCNA and cyclin D1, which are markers of cell proliferation (Figure 2F-G). These results supported the conclusion that knockdown of miR-214-3p alleviated mesangial hypercellularity and renal lesions in IgAN mice.

### Inhibition of miR-214-3p suppressed MCs proliferation

**Figure 3:**
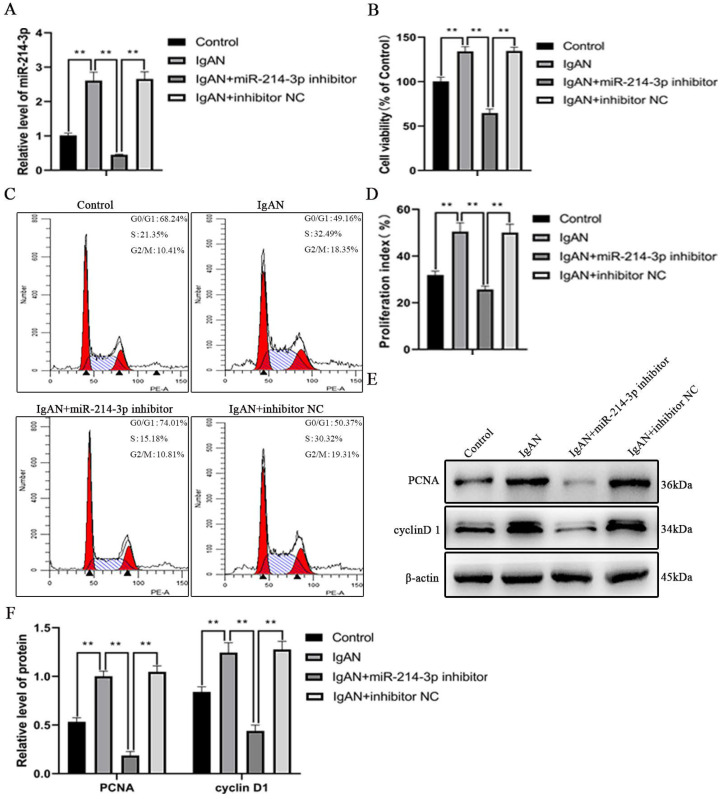
** Inhibition of miR-214-3p suppressed mesangial cell (MC) proliferation.** MCs were transfected with miR-214-3p inhibitor or negative control (inhibitor NC) and then treated with 25 µg/mL p-IgA1 for 24 hours. (A) The expression of miR-214-3p in MCs. The relative level of miR-214-3p was normalized to the expression of U6, and the ratio of control mice was arbitrarily set as 1. (B) Cell viability was measured by CCK8 assay. (C-D) Cell cycle analysis was performed by staining DNA with propidium iodide prior to flow cytometry. The proliferation index (PI) was calculated as the sum of the percentage of cells in S phase and G2/M phase. (E) Immunoblot analyses and (F) quantitative determination of PCNA and cyclinD1 protein levels in MCs. For densitometry, the signals of the target proteins were normalized to the β-actin signal of the same samples to determine the ratios. All data are expressed as the mean ± SD. *P < 0.05, **P < 0.01.

As shown in Figure 3A, miR-214-3p expression was upregulated when MCs was stimulated with p-IgA1 to promote the IgAN pathological state, but was significantly downregulated after transfection with the miR-214-3p inhibitor. The CCK-8 assay indicated that p-IgA1 increased cell viability, while the miR-214-3p inhibitor blocked this promotion (Figure 3B). To further confirm the effect of miR-214-3p on the proliferation of MCs, we performed cell cycle analysis by flow cytometry. p-IgA1 increased the proportion of cells in S and G2/M phases after 24 hours of incubation. With the application of the miR-214-3p inhibitor, more cells were arrested in G1 phase (Figure 3C-D). In addition, the expression of cell proliferation markers, such as PCNA and cyclin D1, was upregulated in IgAN. Transfection with the miR-214-3p inhibitor reduced PCNA and cyclin D1 protein levels (Figure 3E-F). Taken together, the above results demonstrated that knockdown of miR-214-3p could inhibit the proliferation of MCs in IgAN.

### Identification of PTEN as a functional target of miR-214-3p

**Figure 4:**
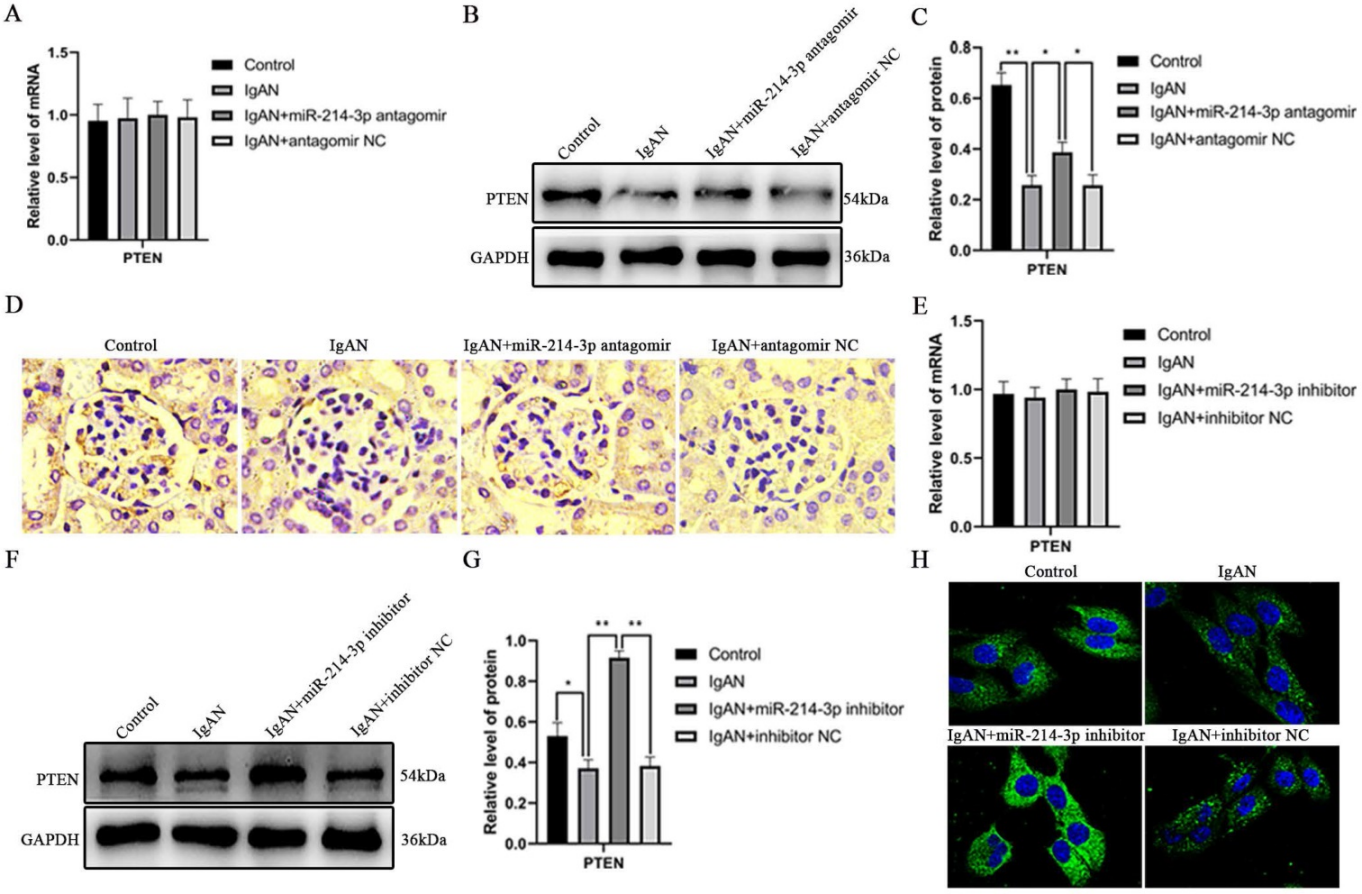
** The effect of miR-214-3p on PTEN expression in IgAN.** (A) PTEN mRNA levels in the mouse renal cortex were determined by real-time PCR. PTEN levels were normalized to GAPDH, and the ratio of control mice was arbitrarily set as 1. (B) Immunoblot analyses and (C) quantitative determination of PTEN protein expression in the renal cortex. For densitometry, the PTEN protein signal was normalized to the GAPDH signal of the same sample to determine the ratio. (D) Immunohistochemical staining illustrated the repressive effect of miR-214-3p on PTEN expression in glomeruli. (E) PTEN mRNA levels in mesangial cells (MCs) were determined by real-time PCR. PTEN levels were normalized to GAPDH, and the ratio of the control group was arbitrarily set as 1. (F) Immunoblot analyses and (G) quantitative determination of PTEN protein expression in MCs. For densitometry, the PTEN protein signal was normalized to the GAPDH signal of the same sample to determine the ratio. (H) Immunofluorescence illustrated the repressive effect of miR-214-3p on PTEN expression in MCs. All data are expressed as the mean ± SD. *P < 0.05, **P < 0.01.

To understand the mechanism whereby miR-214-3p contributed to IgAN, we used TargetScan to search for target genes of miR-214-3p. As illustrated in Figure S1A, the 3'-UTR of mouse PTEN mRNA contained a putative miR-214-3p target site. miR-214-3p is conserved in human and mouse. Analysis of the human and mouse PTEN 3'-UTRs revealed highly conserved recognition elements with significant homology at the miR-214-3p seed sequence. To examine whether PTEN was indeed a target of miR-214-3p, we first evaluated the effect of miR-214-3p on PTEN expression. As shown in Figure 4A-C, downregulation of miR-214-3p did not change the levels of PTEN mRNA, but elevated PTEN protein levels in mice. The in-vivo suppressive effect of miR-214-3p on PTEN was further verified by immunohistochemical staining (Figure 4D). Consistently, *in vitro*, although there was no detectable change in PTEN mRNA levels, p-IgA1 induced a decrease in PTEN protein levels, while transfection of miR-214-3p inhibitor markedly increased PTEN protein levels in MCs (Figure 4E-G). Immunofluorescence also confirmed that miR-214-3p inhibited PTEN expression in MCs (Figure 4H). To determine whether PTEN was a direct target of miR-214-3p, we performed a luciferase reporter assay. Wild-type and mutant 3′-UTR luciferase reporter constructs of PTEN were generated (Figure S1B), and these vectors were co-transfected into HEK-293T cells with miR-214-3p mimic or mimic NC, respectively. The results showed that the miR-214-3p mimic significantly decreased the luciferase activity of the wild type reporter, but had no effect on the luciferase activity of the mutant reporter (Figure S1C). These findings demonstrated that miR-214-3p may directly target PTEN mRNA to repress its translation in IgAN.

### miR-214-3p regulated activation of JNK/c-Jun pathway

**Figure 5:**
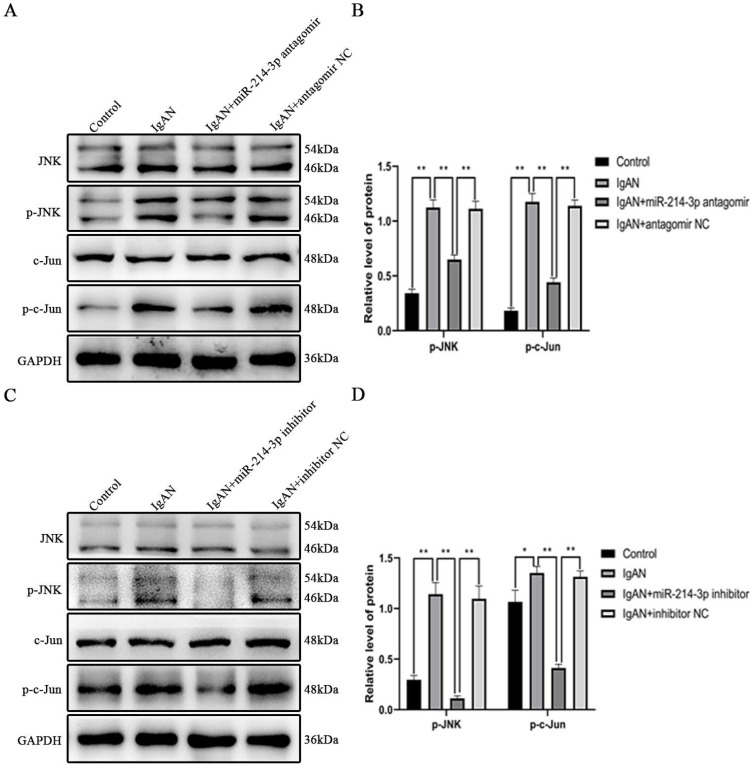
** miR-214-3p regulated activation of the JNK/c-Jun pathway.** (A) Immunoblot analyses and (B) quantitative determination of p-JNK and p-c-Jun protein levels in the renal cortex. (C) Immunoblot analyses and (D) quantitative determination of p-JNK and p-c-Jun protein levels in mesangial cells. For densitometry, the signals of phosphorylated protein were normalized to the corresponding total protein signals of the same samples to determine the ratios. All data are expressed as the mean ± SD. *P < 0.05, **P < 0.01.

PTEN is an endogenous inhibitor of JNK/c-Jun pathway activation 14, 15. Activation of JNK/c-Jun promotes cell proliferation by accelerating G1-S cell cycle progression 16-18. Since we found that miR-214-3p reduced PTEN expression and promoted the proliferation of MCs, we investigated its involvement in the activation of the JNK/c-Jun pathway. *In vivo*, p-JNK and p-c-Jun protein levels were significantly increased in IgAN mice compared with controls. However, treatment with the miR-214-3p antagomir significantly restored the expressions of these proteins in IgAN (Figure 5A-B). *In vitro*, p-IgA1 induced a marked increase in p-JNK and p-c-Jun in MCs. Similarly, inhibition of miR-214-3p suppressed p-JNK and p-c-Jun expression in IgAN (Figure 5C-D). These results verified that miR-214-3p regulated the activation of the JNK/c-Jun pathway in IgAN.

### miR-214-3p targeted PTEN to promote MC proliferation though the JNK/c-Jun pathway in IgAN

**Figure 6:**
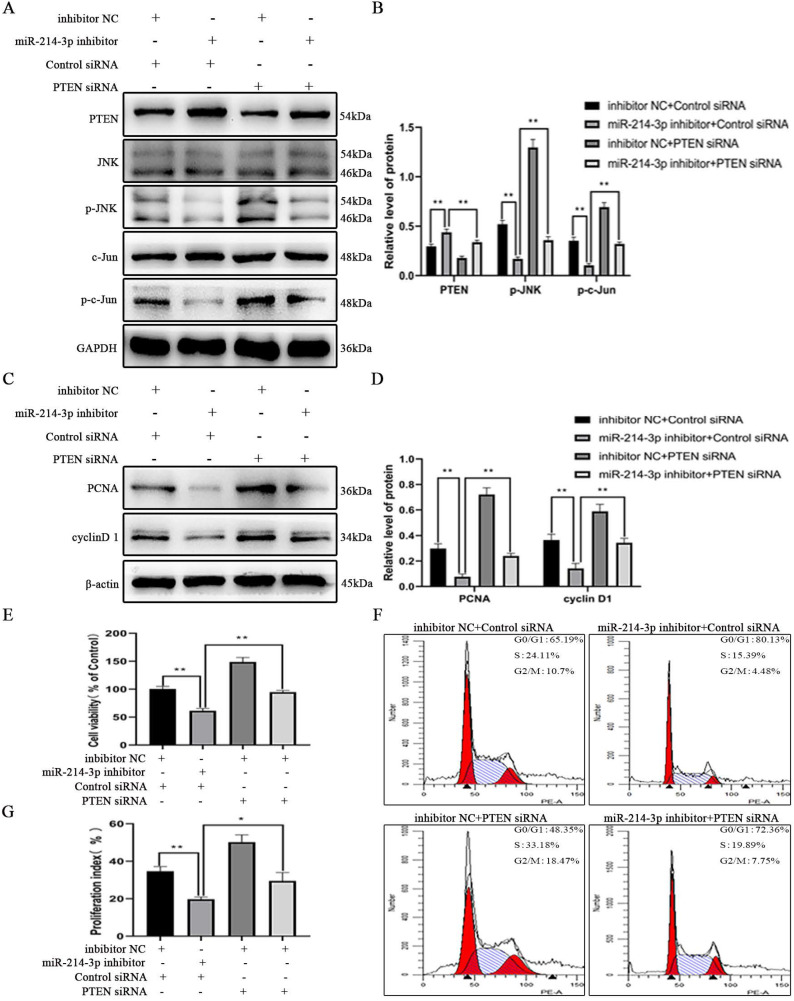
** miR-214-3p targeted PTEN to promote mesangial cell (MC) proliferation through the JNK/c-Jun pathway in IgAN.** MCs were co-transfected with miR-214-3p mimic or negative control (mimic NC) and PTEN siRNA or control siRNA for 6 hours and then treated with 25 µg/ml p-IgA1 for 24 hours. (A, C) Immunoblot analyses and (B, D) quantitative determination of PTEN, p-JNK, p-c-Jun, PCNA and cyclinD1 protein levels. For densitometry, the PTEN protein signal was normalized to the GAPDH signal, the phosphorylated protein signal was normalized to the corresponding total protein signal, and the PCNA and cyclinD1 signals were normalized to the β-actin signal. (E) Cell viability was measured by CCK8 assay. (F-G) Cell cycle analysis was performed by staining DNA with propidium iodide prior to flow cytometry. The proliferation index (PI) was calculated as the sum of the percentage of cells in S phase and G2/M phase. All data are expressed as the mean ± SD. *P < 0.05, **P < 0.01.

To test whether miR-214-3p promoted MC proliferation though the PTEN/JNK/c-Jun signaling pathway in IgAN, MCs was incubated with p-IgA1 and divided into the following four groups: inhibitor NC + control siRNA, miR-214-3p inhibitor + control siRNA, inhibitor NC + PTEN siRNA, and miR-214-3p inhibitor + PTEN siRNA. Immunoblot analysis detected that transient transfection of the miR-214-3p inhibitor increased PTEN expression and reduced the protein levels of p-JNK, p-c-Jun, PCNA and cyclin D1 (Figure 6A-D). CCK-8 assay and cell cycle analysis provided evidence that knockdown of miR-214-3p could inhibit the proliferation of MCs (Figure 6E-G). However, simultaneous co-transfection with PTEN siRNA attenuated all these alterations. These data conclusively suggested that miR-214-3p stimulated the proliferation of MCs by regulating the PTEN/JNK/c-Jun signaling pathway in IgAN.

## Discussion

**Figure 7:**
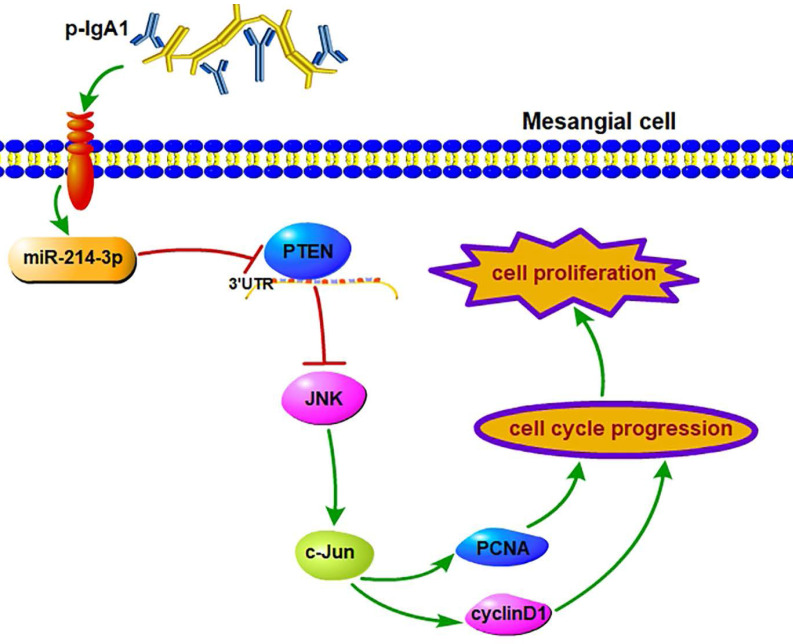
** miR-214-3p is involved in mesangial cell proliferation by targeting PTEN‑mediated JNK/c-Jun signaling in IgA nephropathy.** p-IgA1 upregulated the expression of miR-214-3p, which targets PTEN to activate JNK/c-Jun signaling, thereby promoting mesangial cell proliferation by accelerating G1-S cell cycle progression.

This is the first study to investigate the biological function of miRNAs expressed in the kidney on the proliferation of MCs in IgAN, and we confirmed the following major findings: (1) the differential expression of miRNAs obtained by miRNA sequencing demonstrated that miR-214-3p was dramatically elevated in the kidney in IgAN, and we validated this finding in MCs using both *in vitro* and *in vivo* models; (2) functionally, miR-214-3p promoted MC proliferation by accelerating G1-S cell cycle progression, indicating that miR-214-3p plays an injurious role in IgAN and that inhibition of miR-214-3p could prevent the progression of IgAN; and (3) mechanistically, miR-214-3p might directly target and repress PTEN to activate JNK/c-Jun signaling-associated MC proliferation in IgAN (Figure 7). Collectively, these findings unveiled a novel mechanism of a miRNA involved in the regulation of MC proliferation and provided potential therapeutic targets for IgAN.

Many miRNAs are involved in the development or progression of IgAN 9, 24. miR148b potentially targets C1GALT1 25, miR-98-5p targets CCL3 to decrease C1GALT1 activity 26, and let-7b alters the expression of GALNT2 27 to modulate Gd-IgA1 levels. miR-223 downregulation promoted glomerular endothelial cell proliferation and monocyte adhesion by upregulating importin a4 and a5 in IgAN 28. miRNA expression analysis in IgAN kidney biopsy samples revealed that dysregulated miRNA levels are related to interstitial fibrosis 29, 30 and to the immune response correlated with disease severity and progression 31, 32. In addition to having critical roles in the pathogenesis and progression of IgAN, specific miRNAs in serum and urine have been discovered as potential noninvasive biomarkers for the diagnosis and monitoring of patients with IgAN 32-35. However, the role of miRNAs in the proliferation of MCs in IgAN remains largely unknown. In the present study, we provided compelling evidence to support that miR-214-3p was involved in MC proliferation in IgAN. miRNA sequencing analysis revealed that miR-214-3p was obviously upregulated in IgAN, and we identified this upregulation both *in vitro* and *in vivo*. Moreover, inhibition of miR-214-3p suppressed MC proliferation and arrested G1-S cell cycle progression. In IgAN mice, miR-214-3p antagomir ameliorated mesangial hypercellularity and renal lesions. Accordingly, miR-214-3p downregulation had kidney protective effects in IgAN.

Among the 25 differentially expressed miRNAs found in the sequencing analysis, why did we choose miR-214-3p for further research? Previous studies have verified that miR-214 is extensively expressed in different tissues and cells and exerts different biological effects. Under normal conditions, miR-214 did not affect kidney development or homeostasis, as miR-214 deletion in mice showed normal glomerular and tubular architecture 36. In diabetic nephropathy, the level of miR-214 was significantly increased, and its downregulation attenuated high glucose-induced mesangial and proximal tubular cell hypertrophy 37, 38. It is upregulated in various cancers, including lung, stomach, and ovarian cancers, but downregulated in hepatocellular, colorectal, and renal cancers, and promotes cell proliferation by targeting different genes 39-44. Thus, miR-214 seemed to act in a disease- and cell-dependent manner. In IgAN, the role of miR-214-3p is less understood. Our sequencing results showed that the increase in miR-214-3p expression was more obvious than that of other elevated miRNAs. *In situ* hybridization analysis showed that the level of miR-214-3p was significantly upregulated in MCs of the renal cortex following IgAN. Then, we further confirmed that miR-214-3p is implicated in MC proliferation both *in vitro* and *in vivo*. These findings were consistent with some previous results showing that miR-214 functions as a cell injury factor by promoting cell proliferation.

After selecting miR-214-3p for our study, we started to explore the mechanisms underlying of miR-214-3p-mediated regulation of MC proliferation. PTEN, which is known as a tumor suppressor gene, was identified as a putative target of miR-214-3p in this research. Why did we choose PTEN for this investigation? It has been documented that inactivation of PTEN is the most common genetic lesion in human cancers, and PTEN could contribute to cellular proliferation, apoptosis, migration, and invasion of various cancers. In the kidney, proximal tubule-specific deletion of PTEN in mice induced renal hypertrophy as a result of increased Akt signaling 13. Overexpression of PTEN ameliorated high glucose-induced mesangial and proximal tubular cell hypertrophy and fibronectin expression by regulating Akt activation 37, 38. Kato et al also pointed out that TGF-β activated Akt in diabetic kidneys by inducing miR-216a and miR-217, both of which target PTEN to promote glomerular hypertrophy and matrix expansion 45. PI3K/Akt signaling was elevated in IgAN rats 46 and human MCs stimulated by pIgA1 47. Preincubation of human MCs with wortmannin (a specific inhibitor of PI3K) blocked Akt phosphorylation and pIgA1-induced cell proliferation, thereby ameliorating renal lesions in IgAN 47. In addition, after unilateral nephrectomy in mice with PTEN deficiency, mTORC1/S6K1 signaling was activated in the compensatory enlargement of the remaining kidney 13. In IgAN rats, the mTOR/S6k1 pathway was activated. Rapamycin (a specific inhibitor of mTOR) blocked the activation of mTOR/S6K1 signaling and alleviated MC proliferation and renal damage in IgAN 48. JNK, functions as a target of the tumor suppressor PTEN, and plays a critical role in the growth and survival of tumor cells though the regulation of c-Jun phosphorylation 49, 50. c‐Jun is the founding member and the most potent transcriptional activator in the AP‐1 family, and is involved in cell proliferation by modulating G1-S cell cycle progression 16-18. It has been reported that BMP7 activates JNK to phosphorylate c-Jun, which in turn governs transcription of the AP-1 element to promote G1-S cell cycle progression and nephron progenitor cell proliferation 18. De Borst et al. found that the expression of p-c-Jun in renal tissues of IgAN patients was higher than that of healthy controls 51, but they failed to explore its regulation and function in IgAN. In the present study, we first confirmed that PTEN was a target gene of miR-214-3p by bioinformatics analysis and luciferase reporter assay and further verified this *in vivo* and *in vitro*. Then, we examined the role of miR-214-3p in the activation of JNK/c-Jun signaling. The results showed that inhibition of miR-214-3p significantly limited the expression of p-JNK and p-c-Jun in both MCs and the renal cortex of mice. Finally, *in vitro* studies illustrated that knockdown of miR-214-3p increased PTEN expression and reduced the levels of p-JNK and p-c-Jun, thus inhibiting MC proliferation in IgAN. Furthermore, these changes could be attenuated by co-transfection with PTEN siRNA. Collectively, the above data suggested that miR-214-3p accelerated MC proliferation in IgAN by directly targeting PTEN to modulate JNK/c-Jun signaling. The mechanisms contributing to the upregulation of miR-214-3p in IgAN are the focus of ongoing work within our laboratory.

Inflammation plays an indispensable role in renal damage and the progression of IgAN. The deposition of circulating immune complexes activates MCs, followed by cell proliferation and excessive production of inflammatory factors that promote renal lesions 52. The NLRP3 inflammasome, is a multiprotein complex, that is key to the inflammatory response and closely associated with the pathogenesis of IgAN 53. Activation of the NLRP3 inflammasome requires two steps, namely, priming and activation. The former controls the expression of NLRP3 (an essential component of the NLRP3 inflammasome) and pro-IL-1β (the precursor of IL-1β), while the latter controls caspase-1 activation. NF-κB is a crucial inducer of NLRP3 inflammasome activation. Inactivation of the NF-κB/NLRP3 inflammasome pathway alleviated IgA deposition, MC proliferation and renal lesions in IgAN 54-56. MAPK is also a potent activator of the priming signal of the NLRP3 inflammasome 57. In cultured macrophages, IgA-ICs significantly enhanced the phosphorylation levels of ERK, JNK and p38 MAPK. Two MAPK inhibitors, PD98059 (to ERK) and SP600125 (to JNK), resulted in obviously reduced NLRP3 and pro-IL-1β expression levels compared to those without 58. In IgAN, whether miR-214-3p could mediate activation of the NLRP3 inflammasome by regulating JNK phosphorylation is worthy of further investigation.

In summary, this study revealed the crucial role of miR-214-3p in IgAN. Downregulation of miR-214-3p suppressed activation of JNK/c-Jun signaling by targeting PTEN, thereby inhibiting MC proliferation and alleviating renal lesions in IgAN. Therefore, a therapeutic blockade of miR-214-3p may have satisfying effects and could be used for patients with IgAN in the future.

## Supplementary Material

Supplementary figures.
