# Supplementary material for: Downregulation of miR‑214-3p attenuates mesangial hypercellularity by targeting PTEN‑mediated JNK/c-Jun signaling in IgA nephropathy
Source: Int J Biol Sci. 2021 Jul 31;17(13):3343–55. doi: 10.7150/ijbs.61274 (PMC8416718; doi:10.7150/ijbs.61274)

Supplementary Figure Legends

Figure S1. Identification of PTEN as a direct target of miR-214-3p. (A) Predicted miR-214-3p binding sites and the PTEN 3′-UTR in humans (hsa) and mice (mmu) by TargetScan. (B) Schematic diagram indicating the mutation of the PTEN 3′-UTR. (C) Luciferase activity of HEK-293T cells transfected with either wild-type or mutant 3′-UTR seed sequence of PTEN plus miR-214-3p mimic and mimic NC. All data are expressed as the mean ± SD. NS, not significant (P>0.05), *P<0.05, **P<0.01.

Figure S2. Uncropped western blot films for all blots.

Figure S1

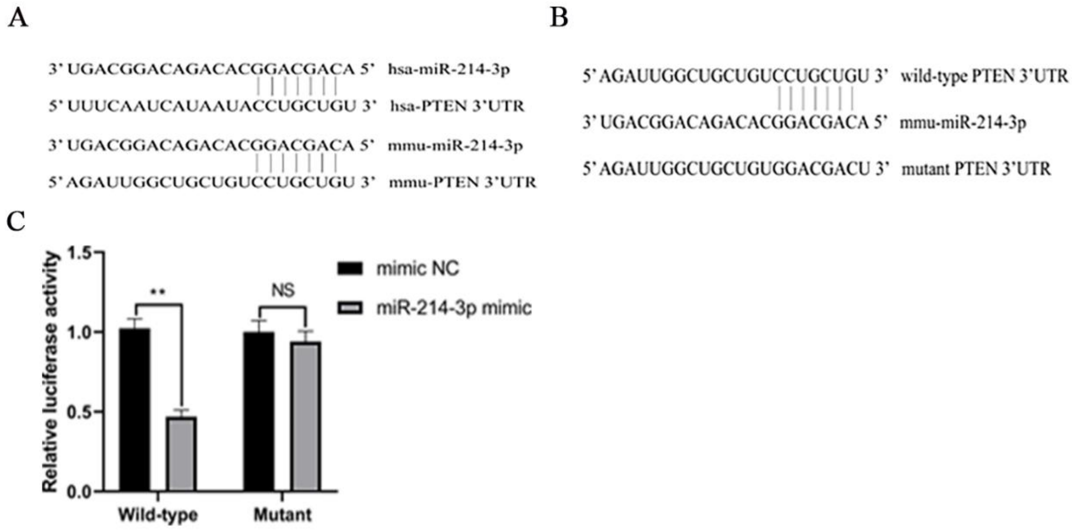
A
3' UGACGGACAGACACGGACGACA 5' hsa-miR-214-3p
5' UUUCAAUCAUAAUACCUGCUGU 3' hsa-PTEN 3'UTR
3' UGACGGACAGACACGGACGACA 5' mmu-miR-214-3p
5' AGAUUGGCUGCUGUCCUGCUGU 3' mmu-PTEN 3'UTR
B
5' AGAUUGGCUGCUGUCCUGCUGU 3' wild-type PTEN 3'UTR
3' UGACGGACAGACACGGACGACA 5' mmu-miR-214-3p
5' AGAUUGGCUGCUGUGGACGACU 3' mutant PTEN 3'UTR
C
Relative luciferase activity
1.5
1.0
0.5
0.0
**
NS
mimic NC
miR-214-3p mimic
Wild-type
Mutant

Figure S2

Figure 2F

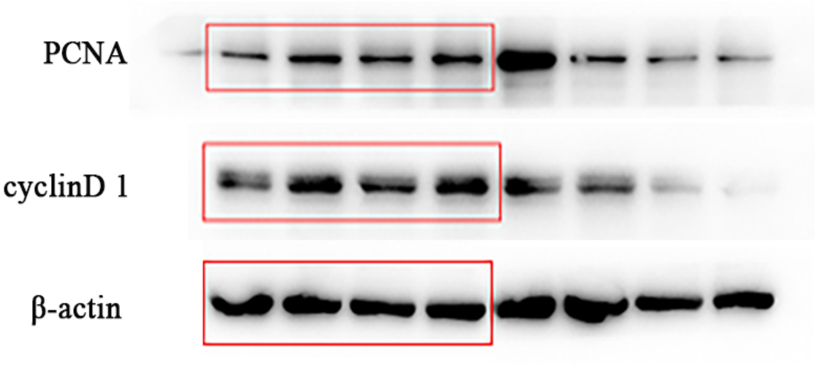

Figure 3E

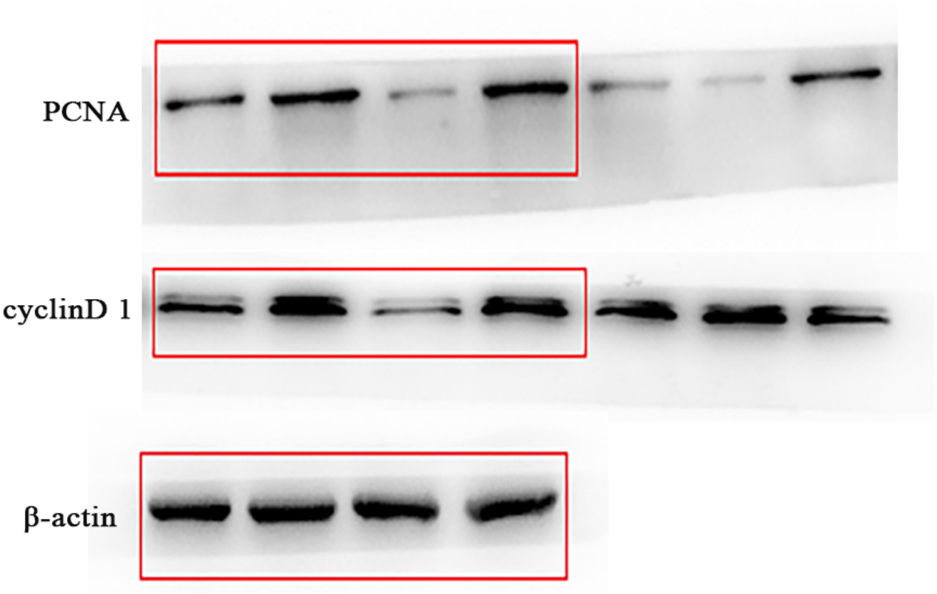

Figure 4C

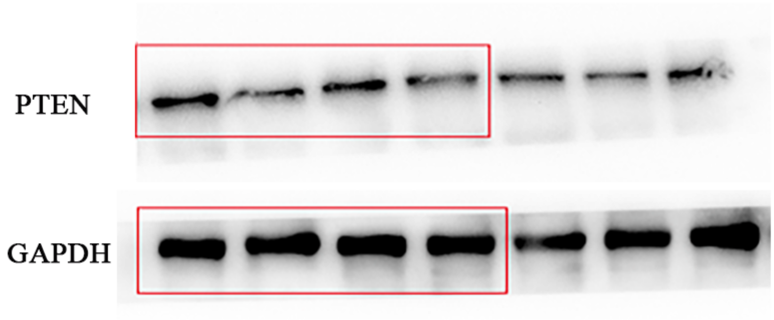

Figure 4G

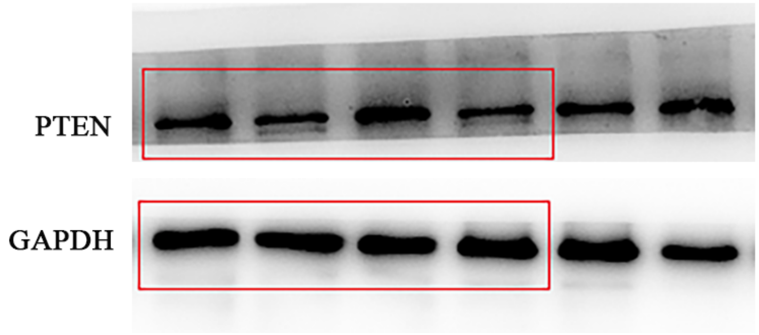

Figure 5A

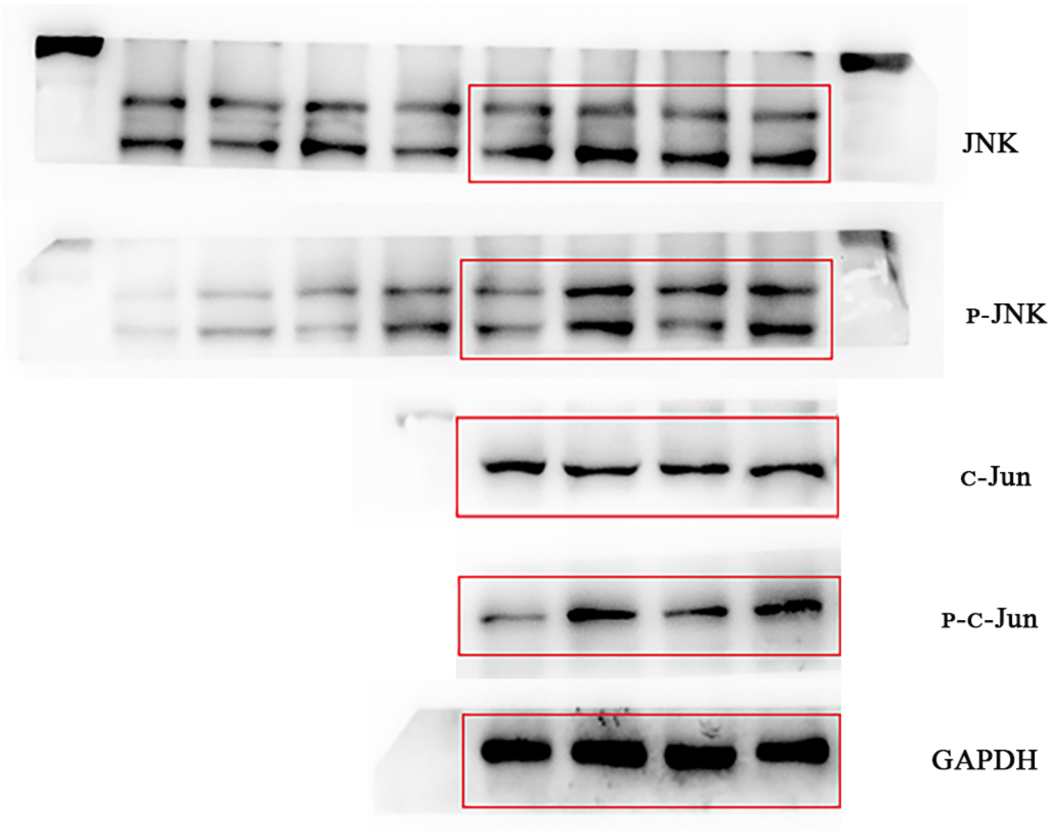

Figure 5C

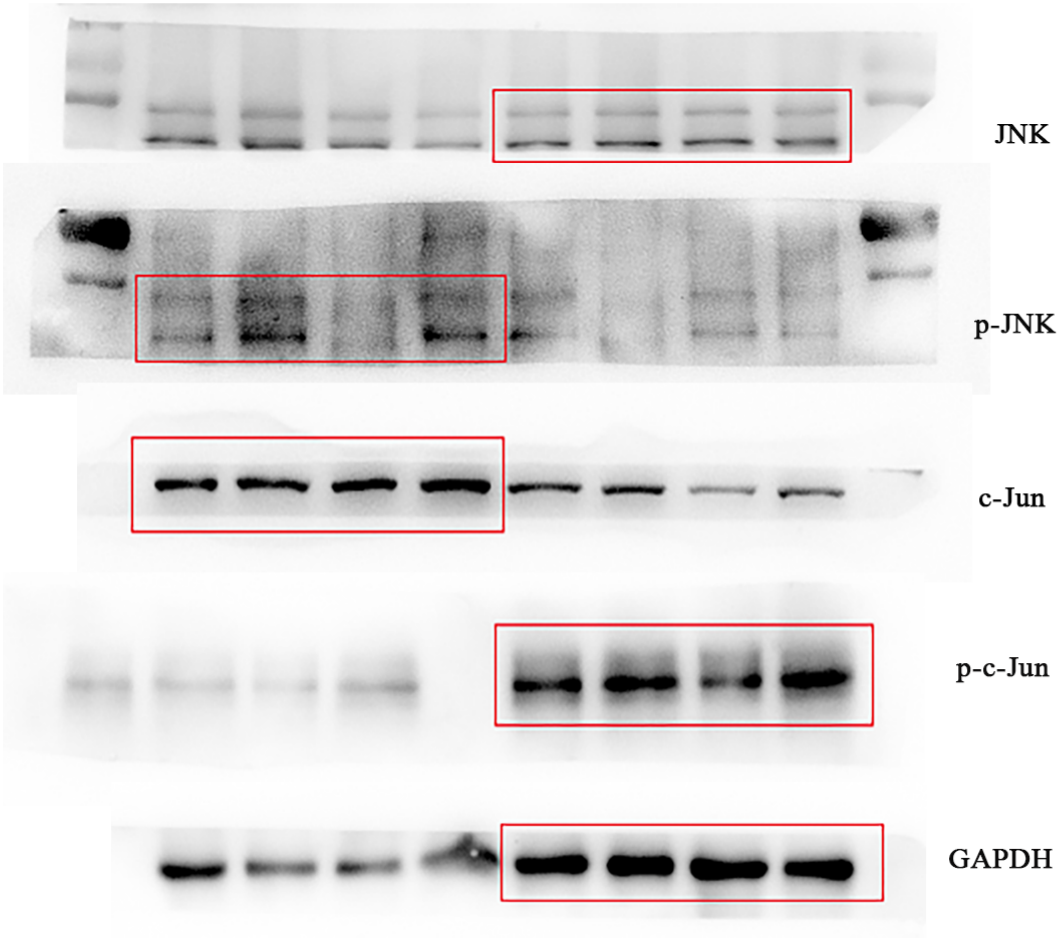

Figure 6A

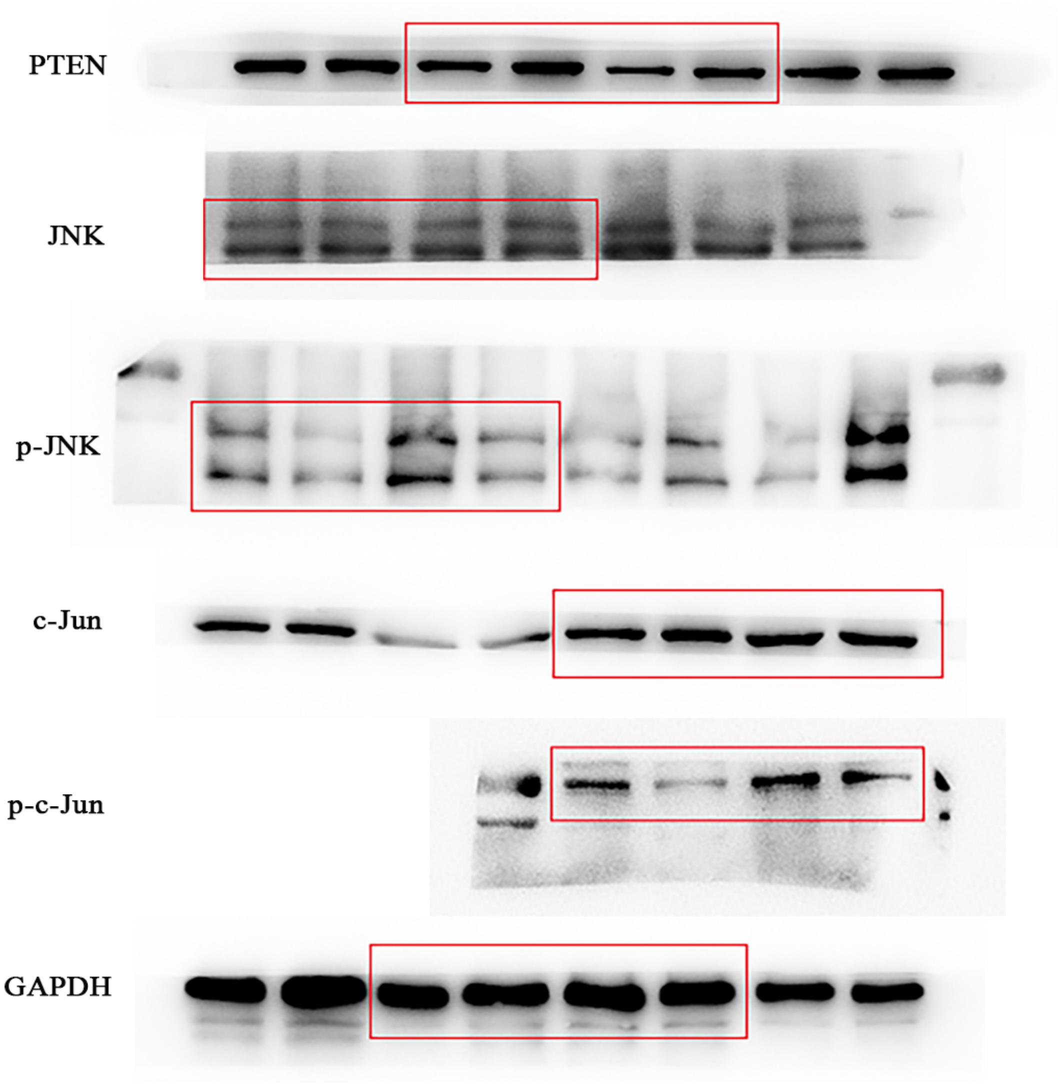

Figure 6C

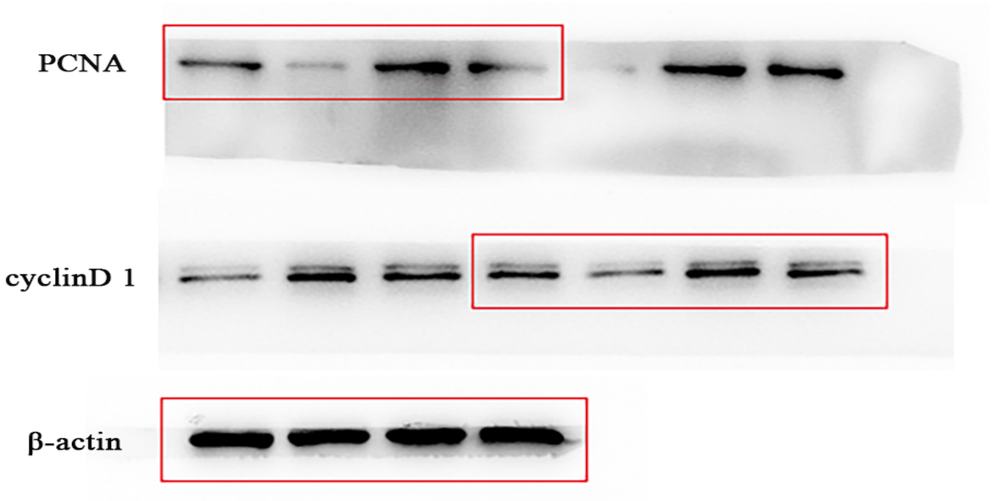

Supplement: Supplementary file 1 — Supplementary figures. [file ijbsv17p3343s1.pdf]
